# Risk and Mitigation of African Swine Fever Virus in Feed

**DOI:** 10.3390/ani11030792

**Published:** 2021-03-18

**Authors:** Megan C. Niederwerder

**Affiliations:** Department of Diagnostic Medicine/Pathobiology, College of Veterinary Medicine, Kansas State University, 1800 Denison Avenue, Manhattan, KS 66506, USA; mniederwerder@vet.ksu.edu

**Keywords:** feed, feed ingredients, trade, African swine fever, foreign animal disease, transmission, virus spread

## Abstract

**Simple Summary:**

African swine fever is the most significant disease threat to swine globally, and recent introductions into previously negative countries has heightened the risk for disease spread. Without an effective vaccine or treatment, the primary objective of negative countries is to prevent African swine fever virus infection in pigs. Significant quantities of feed ingredients used for swine diets are traded worldwide and may be imported from countries with African swine fever. If feed ingredients are contaminated with the virus, they can serve as potential routes for the introduction and transmission of African swine fever virus. This review provides information on the risk of African swine fever virus in feed and the mitigation strategies that may help protect the global swine population from introduction and spread through feed.

**Abstract:**

Since the 2013 introduction of porcine epidemic diarrhea virus into the United States (U.S.), feed and feed ingredients have been recognized as potential routes for the introduction and transmission of foreign animal diseases of swine. Feed ingredients for swine diets are commodities traded worldwide, and the U.S. imports thousands of metric tons of feed ingredients each year from countries with circulating foreign animal diseases. African swine fever (ASF) is the most significant foreign animal disease threat to U.S. swine production, and the recent introduction of ASF into historically negative countries has heightened the risk for further spread. Laboratory investigations have characterized the stability of the ASF virus (ASFV) in feed ingredients subjected to transoceanic shipment conditions, ASFV transmissibility through the natural consumption of plant-based feed, and the mitigation potential of certain feed additives to inactivate ASFV in feed. This review describes the current knowledge of feed as a risk for swine viruses and the opportunities for mitigating the risk to protect U.S. pork production and the global swine population from ASF and other foreign animal diseases.

## 1. Risk of ASFV to the Swine Industry

African swine fever virus (ASFV) is arguably the most significant threat to worldwide pork production due to its high case fatality rate, recent emergence in new countries and continents [1], lack of a commercially available vaccine [2], and substantial impacts on global markets. Importantly, ASF is a trade-limiting disease with significant implications for both global pork and agricultural commodities; economic losses due to ASFV introduction into the United States (U.S.) are estimated to be between $15 and $50 billion, depending on the disease spread in the feral swine population [3].

**Figure 1:**
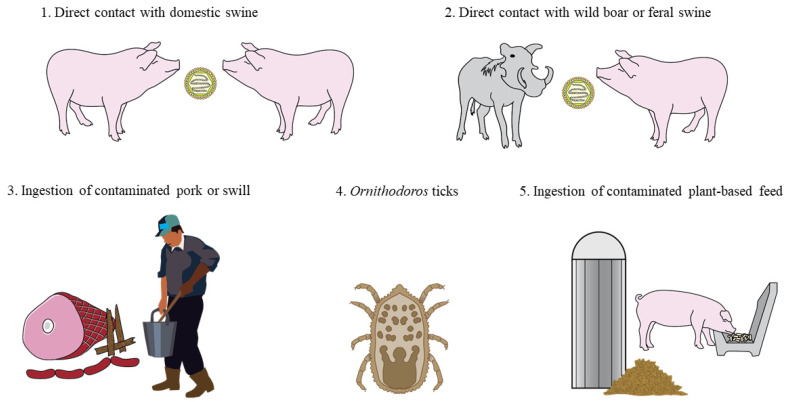
Routes of African swine fever virus (ASFV) transmission. Figure depicts potential modes of ASFV transmission, including (1) direct contact with excretions and secretions from infected domestic swine, (2) direct contact with excretions and secretions from infected wild boar or feral swine, (3) natural oral consumption of uncooked contaminated pork products or swill, (4) vector-borne transmission through the bite of an infected soft tick (*Ornithodoros* spp.), and (5) natural oral consumption of contaminated plant-based feed. Non-depicted modes of ASFV transmission include exposure to contaminated fomites, such as boots, clothing, pens, trucks, and other inanimate materials.

ASFV is an enveloped double-stranded DNA virus in the family *Asfarviridae* [4]. A complex and unique virus, ASFV only infects pigs and presents several distinct challenges to disease control. ASFV is the sole virus classified in the family *Asfarviridae*, which precludes the translation of knowledge on closely related viruses to ASFV pathogenesis and protective correlates. Furthermore, a cursory comparison between ASFV and influenza A virus (IAV) in regard to genome length (170–190 kbp ASFV genome versus 13.5 kb IAV genome) and the number of encoded proteins (151–167 ASFV proteins versus 11 IAV proteins) underscores the complexity of the ASF virus [5,6]. ASFV is highly pathogenic, causing widespread hemorrhage and mortality rates approaching 100% in infected pigs [7]. Transmission routes for ASFV are diverse (Figure 1) and include both direct contact with infected domestic or wild pigs [8] as well as indirect contact with infectious fomites and consumption of contaminated swill or feed [9]. Unique to ASF is the vector transmission through soft ticks of the *Ornithodoros* spp. [10], characterizing ASFV as the lone arthropod-borne virus with a double-stranded DNA genome. ASFV is stable in the environment due to resistance to pH and temperature extremes relative to other swine viruses [11], survives for months in contaminated pork products, and has the potential to become endemic in feral swine [12]. With no commercially available vaccine for preventing infection [13] or treatment available to reduce disease severity in infected pigs, the overwhelming objective of negative countries is to prevent ASFV introduction through biosecurity of people, animals, feed, and supplies entering farms.

ASFV infection and outbreaks in swine were originally described over a century ago in East Africa [14]. In recent years, since the 2007 introduction of ASFV into the Caucasus region of Georgia [15], there has been steady emergence of this virus in new countries and regions that have historically been negative. Examples of regions and countries reporting ASFV introduction over the decade following 2007 include the Russian Federation [16], Poland [17], Latvia [18], and the Czech Republic [19]. On 3 August 2018, the first introduction of ASFV was reported in China, home to the world’s largest population of pigs and pork consumers [20,21]. Over the months following ASFV incursion into China, the virus spread rapidly to at least 12 other Asian and South Pacific countries [1], including Mongolia [22], Vietnam [23], South Korea [24], and Timor-Leste [25]. Concurrent to the spread of ASFV in Asia, dissemination of the virus continued to be reported across several European countries, including Romania [26], Bulgaria [27], Belgium [28], and Serbia [29]. Moreover, Germany, the largest swine producer in the European Union (EU), identified its index case in an adult female wild boar on 10 September 2020 [30].

## 2. Introduction of Feed Risk

Swine enteric coronaviruses, including porcine epidemic diarrhea virus (PEDV) and porcine deltacoronavirus (PDCoV), are considered the last major transboundary swine diseases introduced into the U.S. pig herd in 2013 and 2014, respectively [31,32]. The causative agents of both diseases are single-stranded enveloped RNA viruses of the family *Coronaviridae* [33]. In contrast to the currently circulating novel coronavirus in humans (SARS-CoV-2), these swine coronaviruses are major causes of gastrointestinal disease in pigs and are not a major cause of clinical respiratory signs. Within 8 weeks after the first case of PEDV detection in North America, the virus had spread to most of the major swine producing regions in the U.S. [34]. Within 1 year after PEDV introduction, the virus was responsible for the loss of 10% of the U.S. swine crop, or approximately 7 million pigs [35]. Death due to this disease is devastating as the majority of severely affected pigs and mortalities are neonates within the first few days of life [31]. Importantly, PEDV rapidly expanded into U.S. feral swine populations, with antibody-positive serum samples being detected in wild pigs less than 1 year after virus introduction into domestic swine [36]. The disease eradication challenges posed by the transmission of porcine viruses into feral swine populations cannot be overstated. Almost 8 years after the introduction of PEDV, this virus continues to cause endemic disease in U.S. swine [37] and investigations continue to seek improvement and refinement of protocols for disease control of circulating historical and novel virus variants [38]. 

After the introduction of PEDV into North America, several epidemiological analyses into the introduction and rapid spread across new farms revealed the potential source of the virus as contaminated feed and feed ingredients [34]. First, the genetic sequences of the PEDV strains that emerged in U.S. swine-producing states shared ≥99.5% nucleotide identity with a PEDV strain that had recently circulated in the Anhui Province of China [39]. Based on this analysis, the authors concluded that the country of origin of U.S. PEDV strains was likely China [39], a country from where thousands of metric tons of feed ingredients had been imported into the U.S. [40]. Second, research revealed that PEDV maintained infectivity in several feed ingredients, including soybean meal, exposed to temperature and humidity conditions simulating a 37-day transpacific shipment environment based on historical meteorological data [41]. Third, experiments confirmed PEDV was transmissible through the natural consumption of contaminated plant-based feed [42] and identified a low minimum infectious dose (10^1.7^ 50% tissue culture infectious dose/g (TCID_50_/g)) required for infection through feed [43]. Fourth, PEDV RNA was detected in feed and feed supplement samples that had been implicated as potential sources of the virus introduction on new farms in Ohio and Canada [44,45]. Retrospective Canadian analyses identified that the receipt of feed from a specific company increased the likelihood of a porcine epidemic diarrhea (PED) outbreak by 38 times [46] and that PED cases were associated with a single feed supplier network [47]. Taken together, feed as a novel risk factor for viral disease introduction on swine farms was recognized due to the collective North American experience with PEDV. Furthermore, epidemiological investigations after PEDV was introduced into other Asian countries corroborated a potential role of feed, such as feed truck deliveries in Japan [48] and feed mill density in Taiwan [49]. 

**Figure 2:**
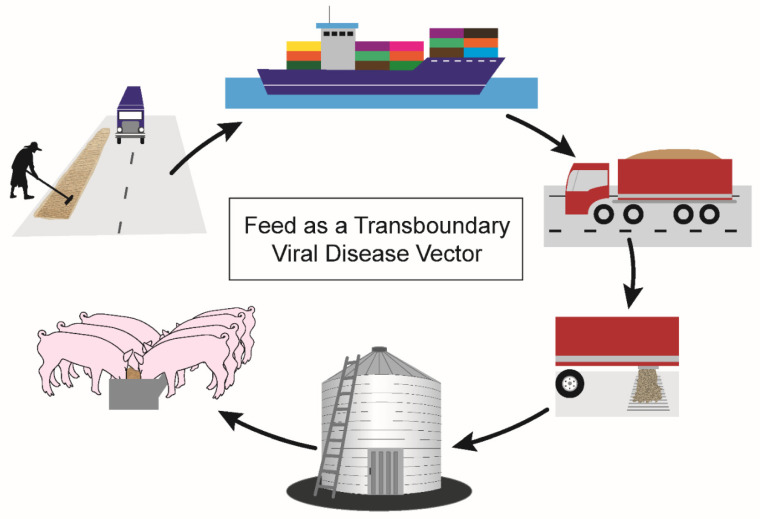
Mechanism by which feed ingredients may serve as transboundary vectors of swine viral diseases. Risky agricultural practices, such as drying grains on roadways, may contaminate feed ingredients in countries with circulating foreign animal diseases. Trucks carrying pigs may drive over areas where feed ingredients are drying, transferring viruses through pig excretions and secretions. Feed ingredients are transported in shipping containers across transoceanic conditions to arrive in the importing country. Trucks transport the feed ingredients on interstate highways to feed mills, where they are incorporated into a complete feed diet. Feed mills widely distribute feed to swine farms for pigs’ consumption and growth.

For feed ingredients to serve as transboundary vectors for viral diseases such as PED and ASF (Figure 2), feeds or ingredients must first have a source of virus contamination. Contamination risks are present at several critical control points during feed manufacture and may be pervasive in countries with uncontrolled outbreaks where widespread environmental contamination has occurred. Specific examples of contamination risks include exposure of pre-harvest field crops to infected wild boar, exposure of post-harvest grains drying on roadways to vehicles transporting infected pigs, exposure of feed-ingredient-processing facilities to infectious fomites such as personnel shoes, exposure of ingredients post-processing to infectious fomites such as multi-use containers, and exposure of stored ingredients to infectious pests. After contamination at any of these control points, feed ingredients from ASF-positive countries would undergo transoceanic shipment across the Atlantic or Pacific Ocean in large shipping containers. Ingredients arrive in bulk at seaports for inspection by U.S. Customs and Border Protection prior to transfer onto trucks for land transport. Finally, feed ingredients arrive at feed mills across the U.S. for inclusion in complete feed diets and delivery to swine farms for consumption (Figure 2). 

Although there are other risk factors, such as illegally smuggled pork products, for introduction of ASFV into the U.S. [50], plant-based feeds and feed ingredients are of particular concern due to several unique characteristics. Concerning aspects of feed ingredients include their global sourcing, intended purpose for pig consumption, distinct access to commercial swine in high-biosecurity farms, and widespread distribution from centralized feed mills. An example of the former characteristic was reported in a 2018 inventory when one U.S. swine farm declared feed ingredients had been sourced from 12 different countries across three continents [51]. Further, the latter characteristic negates the need for farm-to-farm proximity, which is important for other introduction routes, such as aerosol, equipment, and personnel. For example, in modeling the direct and indirect sources of PEDV spread over 5 months within one U.S. production system, VanderWaal et al. (2018) reported feed as a more often attributed transmission source between physically distanced farms [52]. Additional data support the concept of feed as a virus source for long-distance spread. Specifically, a U.S. survey identified feed as the PEDV source more often in regions considered non-swine-dense [34], and a Japan survey identified feed truck visits as a risk for PEDV introduction on only those farms located greater than 5 km from other infected farms [48]. Furthermore, a recent real-world demonstration project reported PEDV survival in feed ingredients transported in a commercial trailer for 21 days over 14 states covering 9741 km [53]. In summary, insight garnered from PEDV has revealed the vulnerability of swine farms to transcontinental and cross-country virus spread through contaminated feed.

Once feed and ingredients were identified as novel routes for transboundary viral disease spread, defining feed risk for other foreign animal diseases (FAD), including ASFV, emerged as a priority for the U.S. swine industry [54]. Historical introductions and spread of ASFV into new countries or regions have had epidemiological associations with contaminated feed in the absence of swill or infectious food waste containing pork. For example, on Romanian backyard farms, one of the risk factors for ASFV incursion during May–September 2019 was feeding plant material that had originated from ASFV-positive regions to pigs [26]. Another example includes Latvia, where contaminated grass and crops fed to pigs were implicated in the 2014 outbreaks of ASFV on backyard farms [18]. Contamination of cereal grains and grasses fed to commercial pigs was also a likely route of ASFV introduction on farms in Estonia between 2015 and 2017 [55]. Further, dried blood products contaminated with ASFV and used as feed additives were suggested contributors to disease spread in China [56,57].

## 3. Certain Ingredients Support ASFV Stability

Identifying which feed ingredients provide an environmental matrix that supports ASFV stability is an important step in determining risk. To evaluate this risk using a transboundary shipment model, Dee et al. (2018) selected 12 feeds, ingredients, or products of animal origin based on import volume and use in swine feed for evaluating ASFV stability [58]. Ingredients included conventional soybean meal, organic soybean meal, soy oilcake, distillers dried grains with solubles (DDGS), lysine, choline, vitamin D, moist cat food, moist dog food, dry dog food, pork sausage casings, and complete feed. Following inoculation with ASFV Georgia 2007, ingredients were exposed to fluctuating temperatures and humidity that replicated real-world meteorological conditions during transoceanic shipment. Ingredients were tested for the presence of infectious virus at the conclusion of the simulated shipment model. After 30 days of transatlantic shipment conditions, ASFV Georgia 2007 was broadly stable across diverse ingredients, with infectious virus being detected in 75% (9/12) of the tested ingredients, including conventional soybean meal, organic soybean meal, soy oilcake, choline, moist cat food, moist dog food, dry dog food, pork sausage casings, and complete feed [58]. 

Importantly, several of the ingredients identified as supportive to ASFV also provided an environmental matrix that stabilized other diverse viruses of concern to swine health. Of the 14 viruses that have been tested in transoceanic shipment models to date [40], 9 (9/14; 64%) and 6 (6/13; 46%) viruses survived the 30 or 37-day environmental conditions in conventional soybean meal and pork sausage casings, respectively. Furthermore, conventional soybean meal and pork sausage casings promoted survival of the four viruses considered the highest priority for preventing entry by the U.S. swine industry [59], including foot-and-mouth disease virus (tested by surrogate Seneca virus A (SVA)), ASFV, classical swine fever virus, and pseudorabies virus. Other ingredients supporting wide-ranging pathogen stability included lysine (6/14; 43%), choline (5/14; 36%), vitamin D (5/14; 36%), and complete swine feed in meal form (5/13; 38%) [40].

ASFV half-lives in feed ingredients provide additional evidence as to the relative virus stability across different matrices. The half-life is independent of titer and is defined as the time necessary for the virus quantity to be reduced to half its initial concentration [60]. The half-life of ASFV Georgia 2007 has been determined in nine feeds and feed ingredients that promoted viral stability in transoceanic shipment conditions [61]. To calculate ASFV half-lives in the nine feed ingredients, viral decay was quantified throughout the 30-day transoceanic model incorporating moderate temperature (mean 12.3 °C) and humidity (mean 74.1%) conditions. Half-life estimates across all feed ingredients were between 9.6 ± 0.4 and 14.2 ± 0.8 days, with an average half-life of 12.2 days. Interestingly, the ASFV half-life in organic soybean meal was 3 days greater in length than conventional soybean meal. Further, all nine feed matrices enhanced ASFV stability compared to laboratory media, in which the ASFV half-life was 8.3 ± 0.3 days [61]. Variability in half-life estimates across feed matrices is likely associated with protein, fat, or moisture content; chemical exposure; and ingredient processing. Overall, the feed matrix promotes ASFV stability, and approximately 2 weeks are required for the virus concentration to decrease by half under shipping conditions.

## 4. ASFV Transmission through Plant-Based Feed

ASFV transmissibility through the oral route has been appreciated for a century and was recognized early on as having more variability than parenteral routes of inoculation [14]. Numerous experimental studies have since reported infection rates of historical and modern ASFV isolates after oral, nasal, or intraoropharyngeal ASFV administration (Table 1). However, transmissibility of the modern ASFV Georgia 2007 isolate through natural drinking of contaminated liquid and natural eating of contaminated plant-based feed was only recently characterized [51]. For this study, infectivity rates in nursery pigs were determined at various ASFV doses consumed naturally in small volumes of liquid media (100 mL) or complete feed in meal form (100 g). Confirmed infection occurred through both drinking and eating routes, with lower doses required for transmission in liquid compared to feed. Specifically, the minimum infectious dose (MID) of ASFV Georgia 2007 in liquid was 10^0^ 50% tissue culture infectious dose (TCID_50_), whereas 10^4^ TCID_50_ was the dose necessary for infection in feed. Statistical modeling of repeated exposures to small volumes over time (i.e., consuming a contaminated batch of feed or drinking contaminated water) revealed an increased likelihood of infection as the number of exposures or total consumption volume rises. Taken together, ASFV is orally transmitted through natural consumption of contaminated plant-based feed, with the infection probability dependent on the quantity of virus present and the volume of feed consumed [51].

Additional studies have published the ASFV dose required for infection through oronasal administration or consumption of the virus in various feed and liquid matrices (Table 1). For example, Pietschmann et al. (2015) reported that doses as low as 10^0.5^–10^1.4^ hemadsorbing units (HAU) of ASFV Armenia 2008 were capable of causing infection through oronasal administration [62]. Inoculation experiments using the ASFV Malawi 1983 isolate determined that a 10^2^ 50% hemadsorbing dose (HAD_50_) was sufficient to cause infection when delivered intranasopharyngeally but not when delivered intraoropharyngeally [63]. When the virus was consumed in cow milk, a dose of 10^5^ HAD_50_ was required for infection with ASFV Tanzania KWH/12 [67]. Blázquez et al. (2020) reported a lack of infection after repeated consumption of ASFV Georgia 2007 (10^4.3^ or 10^5^ TCID_50_) mixed in liquid porcine plasma and complete feed [68]. Early studies on pathogenesis also highlighted infection variability through the oral route, including a lack of infection after ingestion of the ASFV East African strain (10^3.7^–10^6.1^ HAD_50_) in liquid or moistened solid feed [66] and successful ASFV infection after ingestion of the ASFV Hinde WHII strain (10^7^–10^7.5^ HAD_50_) in dry feed [71]. Furthermore, Montgomery (1921) described infection of pigs through consumption of infectious feces- or urine-contaminated feed, while consumption of ASFV in sweet potatoes or bananas failed to result in infection [14]. These studies underscore the importance of ingredient composition when considering feeds and liquids as delivery vehicles for ASFV via the oral route.

## 5. Reducing ASFV Risk through Feed Biosecurity

**Figure 3:**
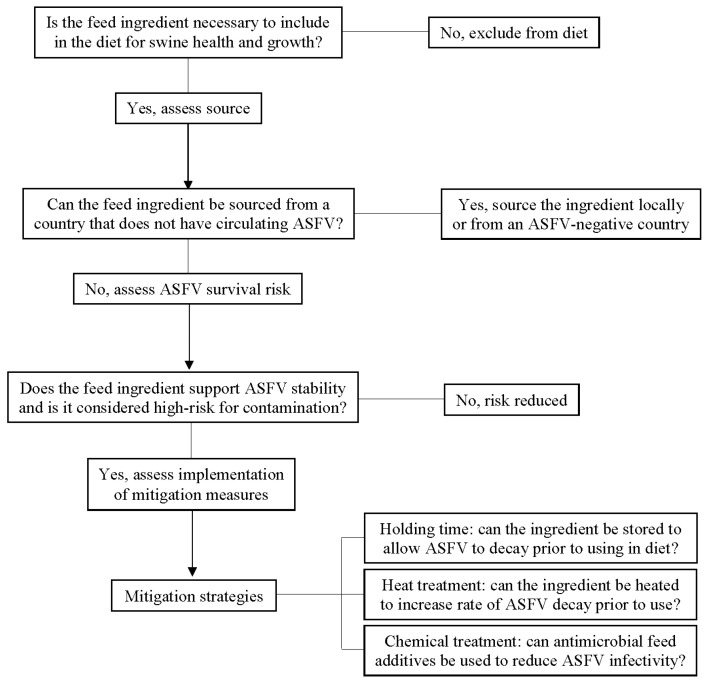
Risk assessment for feed ingredients as transboundary vectors of swine viral diseases. Adapted and modified from the Feed Ingredient Safety Decision Tree Matrix [75].

As a relatively new area of specialization in the biosecurity realm, feed biosecurity has become an important and widely recognized biosecurity target critical for the prevention of porcine viral disease entry onto farms. When examining feed ingredients as a potential pathogen source, several factors influence this biosecurity risk [73]. Assessment of risk starts with characterizing the necessity, source, and virus stability data of each feed ingredient (Figure 3). First, inclusion of the ingredient should be confirmed necessary for swine health and growth, and it should lack a suitable, cost-effective, and lower-risk alternative. Second, the disease status of the country of origin for each ingredient should be considered, including swine disease outbreaks in specific regions or endemic diseases of widespread prevalence. For instance, sourcing feed ingredients from the U.S. currently poses no risk for ASFV introduction but does not eliminate the possibility of feed as a vector for currently circulating diseases such as PED. Moreover, risks in positive countries across the world may vary depending on the disease epidemiology at the time of ingredient manufacture and import. For example, when considering soy-based feed ingredients imported to the U.S. from ASFV-positive countries in 2018 and 2019, the greatest volume was received from China and Ukraine [74], two countries with very different epidemiological situations impacting risk.

As a third consideration, the environmental stability of the virus in the feed ingredient plays a role in risk. Specifically, experimental research has identified high-risk ingredients, such as conventional soybean meal, which provide environmental matrices conducive to broad and diverse pathogen stability across DNA and RNA as well as enveloped and non-enveloped viruses [40]. Ingredients that provide widely supportive environments are risks for those pathogens not yet tested as well as those pathogens yet to emerge. On the other hand, certain types of pathogens (e.g., non-enveloped viruses) are generally stable across most environments. Finally, the agricultural or manufacturing practices used to produce the ingredient impact risk. For example, the practice of drying grains on roadways shared by trucks transporting live swine increases the chance of viral contamination. In contrast, ingredients manufactured and sealed in biosecure facilities with safe processes and a low likelihood of environmental exposure pose fewer risks.

Feed, ingredient, and feed mill biosecurity is essential for reducing infectious disease risks at all stages of swine production [76,77], and implementation of biosecurity procedures focused on feed can help address these risks. Breaches in feed biosecurity can result in virus contamination during the growing, harvesting, processing, or post-processing of crops intended for swine feed. In a review of the prevention of ASFV incursion onto EU backyard farms, recommendations included not providing pigs any newly harvested feedstuff from regions with ASFV [78]. This recommendation was driven by epidemiological investigations linking fresh grass and seeds contaminated by secretions of infected wild boar with access to fields [79]. Further, ensuring the biosecurity of receptacles for feed transport is essential, as contaminated flexible intermediate bulk containers were implicated as a likely root cause for the U.S. PEDV outbreak [80]. Finally, secure storage of feed intended for pig consumption is necessary, as unsafe feed stores have been associated with ASFV introduction on farms in the EU [55].

Recently, the Canadian Food Inspection Agency (CFIA) sought to develop risk assessment criteria for livestock feed mills and published 34 risk factors identified as important for feed mill safety and security. Among the identified factors were manufacturing practices allowing feed contamination through open equipment; use of at-risk imported feed ingredients; control measures for incoming ingredients, such as analysis certificates or supplier audits; and controls for finished feed, such as single-use packaging material and transport sequencing [81]. Many current biosecurity protocols for swine farms can be directly translated to the feed mill environment. Protocols may include regulations on (1) limiting access of people and vehicles, (2) showering prior to facility entry, (3) changing of clothes and shoes prior to entry, (4) forming lines of separation or barriers to identify restricted areas, (5) prohibiting high-risk product entry, (6) disinfecting supplies and equipment, (7) ensuring cleaning and hygiene of staff, (8) ensuring quarantine time for employees and visitors traveling to ASFV-positive countries, (9) limiting personnel exposure to swine, (10) performing pest control, (11) decontaminating transport and delivery vehicles, and (12) providing training on safe feed handling for mill operators and truck drivers. Reported by Pudenz et al. (2019), biosecurity practice adoption is impacted by swine producer demographics, operation type, and feasibility of implementation. Fortunately, feed biosecurity procedures included in the Secure Pork Supply Plan, such as receiving and storing feed in pest-resistant containers and sweeping up spilled feed, were reported to be highly adopted by over 90% of the >300 surveyed producers in the U.S. [82]. 

## 6. Physical Mitigation Methods for ASFV in Feed 

In addition to biosecurity and sourcing considerations, physical and chemical treatments of feed or ingredients can be tools for risk mitigation of ASFV. Implementing feed quarantine [54], or storage of ingredients after import from high-risk countries and regions, is one strategy intended to allow virus decay prior to incorporation of the ingredients into swine diets. For example, ASFV half-lives [61] were recently used to provide holding time information to U.S. swine producers for 99.99% degradation of ASFV in high-risk feed ingredients [83]. Holding times were based on 13 half-lives, which is the time required to reduce the ASFV concentration to 0.01% of its initial quantity. Mean holding times ranged between 125 and 168 days for conventional soybean meal, organic soybean meal, and choline exposed to moderate environmental conditions at a mean temperature of 12.3 °C. Further, holding times were reported for 99.99% degradation of SVA in conventional soybean meal, DDGS, vitamin D, and lysine at three mean temperatures. Holding times ranged from 39 to 494 days at 4 °C, 13 to 182 days at 15 °C, and 13 to 26 days at 30 °C [83].

In March 2019, the CFIA implemented storage requirements for unprocessed grains, oilseeds, and associated meals imported from countries at risk for ASFV contamination. These storage regulations were intended to mitigate the risk of ASFV introduction into Canada through imported plant-based feed ingredients. Regulations included storage of ingredients for at least 100 days at 10 °C or 20 days at 20 °C. Alternatively, the CFIA provided regulatory guidelines for heat treatment of feed ingredients to increase the rate of viral decay and further reduce the risk of ASFV. Specifically, feed ingredients are heated for 30 min at 70 °C or 5 min at 85 °C. Storage time or heat treatments are required prior to imported products entering the livestock feed chain [84]. In the EU, where ASFV is present in wild boar and contamination of field crops has been reported, recommendations include storing fresh grass and grains for 30 days prior to feeding and storing straw for 90 days prior to bedding use. These recommendations are to reduce the risk of field crops being a source of ASFV for local pig farms [85].

Heat treatments and storage of crops and plant-based ingredients have demonstrated experimental efficacy in reducing the infectivity of swine viruses such as ASFV and PEDV. For example, Fischer et al. (2020) contaminated field crops, including wheat, barley, rye, triticale, corn, and peas, with ASFV Armenia 2008 prior to subjecting the crops to a 2-h drying period at 20 °C. After 2 h of storage at room temperature, no infectious virus could be isolated from the unprocessed crops [86]. Stability of an ASFV isolate from the Russian Federation was investigated at various temperatures in compound feed made primarily of barley and wheat [87]. Results reported that infectious ASFV was undetectable in the inoculated feed after 5 days at 22–25 °C and after 40 days at 4–6 °C. At temperatures between –16 °C and –20 °C, infectious ASFV was detectable in the plant-based compound feed for the entire length of the 60-day study [87]. 

Trudeau et al. (2017) reported the stability of PEDV, PDCoV, and transmissible gastroenteritis virus (TGEV) at 25 °C in porcine complete feed and several ingredients, including spray-dried porcine plasma, meat meal, meat and bone meal, blood meal, corn, soybean meal, and DDGS. At the conclusion of the 56-day study, infectious PEDV, PDCoV, and TGEV were still detectable in all tested feeds and ingredients, with soybean meal maintaining the highest titer of all three viruses [88]. Other work by the same group investigated the heat treatment of PEDV-contaminated complete feed, reporting virus inactivation in the feed after 25 min at 120 °C, 15 min at 130–140 °C, and 10 min at 145 °C [89]. Further, significant titer reductions of PEDV in nine different contaminated feeds and ingredients were reported after heat treatment using lower temperatures: after 30 min, a 2.4 log reduction at 60 °C, a 2.7 log reduction at 70 °C, a 3.4 log reduction at 80 °C, and a 3.9 log reduction at 90 °C [90].

## 7. Chemical Mitigation Methods for ASFV in Feed

Feed additives with antimicrobial activity against ASFV and other swine viruses have gained substantial interest in the wake of feed risk awareness and the need for antibiotic alternatives [91]. Studies evaluating the efficacy of various chemical feed mitigants are summarized in Table 2. Primary additive classes investigated for antiviral activity include aqueous formaldehyde, medium-chain fatty acids, short-chain fatty acids, organic acids, and essential oils. Mechanistically, these antimicrobial products inactivate viruses in different ways and regulations on use vary by country. For example, medium-chain fatty acids (MCFA) are believed to reduce virus infectivity by disrupting the viral envelope, leading to deconstruction of the virion and an inability to bind to the host cell for entry [92]. A second example is aqueous formaldehyde, which is believed to reduce virus infectivity through alkylation and cross-linking of viral nucleic acids and proteins [93].

The efficacy of both MCFA and aqueous formaldehyde has been experimentally confirmed for ASFV. In Niederwerder et al. (2020), MCFA (1:1:1 ratio of C6, C8, and C10) and aqueous formaldehyde (Sal CURB^®^) were investigated for their ability to inactivate or reduce the infectivity of ASFV in cell culture and in feed under a transoceanic shipment model [94]. In cell culture, dose–response curves were determined by adding MCFA or aqueous formaldehyde at various inclusion rates (0.03–2.0%) to a standard volume of ASFV; titration assays were performed to quantify ASFV remaining post-exposure to liquid additives. Results demonstrated a dose-dependent reduction in the ASFV titer after exposure to either product, with inclusion rates defined for MCFA (0.70%) and formaldehyde (0.35%) required to reduce ASFV below the level of detection in cell culture. In the transoceanic model, MCFA and aqueous formaldehyde were tested against ASFV in nine different feed ingredients: conventional soybean meal, organic soybean meal, soy oilcake, choline, moist dog food, moist cat food, dry dog food, pork sausage casings, and complete feed in meal form. ASFV-contaminated ingredients were mixed with either MCFA (1.0% inclusion) or aqueous formaldehyde (0.33% inclusion) during the 30-day model simulating shipment conditions. Although all treated feed ingredients maintained detectable ASFV DNA on PCR, results demonstrated reduced ASFV infectivity post-treatment, with most MCFA-treated feed ingredients (16/18) and all formaldehyde-treated feed ingredients (18/18) lacking infectious ASFV at the conclusion of the study [94]. Under the conditions of these studies, both MCFA-and formaldehyde-based feed additives demonstrated efficacy in a dose-dependent manner for reducing ASFV infectivity and show potential as mitigation tools for reducing the risk of ASFV introduction and transmission through feed.

Several additional studies have confirmed the antiviral effects of MCFA, aqueous formaldehyde, organic acids, and other additives against swine viruses endemic to the U.S. (Table 2). Various testing methods have included culturing in vitro on cell lines, storing at various time and temperature combinations, exposing to transoceanic shipment conditions, flushing feed manufacturing equipment, and feeding through natural consumption. For example, Dee et al. (2020) reported a robust analysis of 15 chemically diverse feed additives for their efficacy against porcine reproductive and respiratory syndrome virus (PRRSV), PEDV, and SVA through the natural consumption of contaminated complete feed. Interestingly, all but one product (14/15; 93%) provided beneficial effects in terms of the outcome, including reduced clinical signs, decreased virus detection in biological samples, and increased average daily gain [96]. Taken together, both physical and chemical treatments provide opportunities to reduce virus risks in feed; however, it is important to note that most methods of mitigation do not eliminate ASFV DNA or other viral nucleic acid from feed, underscoring the importance of determining virus biological infectivity after mitigation is applied.

## 8. Conclusions

Experimental research has proven that ASFV is broadly stable across commonly imported feed ingredients, transmission is possible through consumption of ASFV-contaminated plant-based feed, and physical and chemical treatments of feed may mitigate the risk of ASFV introduction. Epidemiological evidence has linked contaminated feed with ASFV field outbreaks in both Europe and Asia. An expanding geographic distribution of ASFV continues to increase the risk of U.S. incursion. With economic losses of ASFV introduction into the U.S. swine herd estimated at >$15 billion due to production losses and market disruption, the importance of preventing entry cannot be overstated. As thousands of metric tons of swine feed ingredients are imported each year into the U.S. from countries with active ASF outbreaks, it is critically important that mitigation strategies be investigated and adopted to reduce the risk of ASFV entry through this route. 

## Figures and Tables

**Table 1:** Infection rates of different doses and strains of African swine fever virus (ASFV) when administered through the oronasal route or consumed through natural eating and drinking behaviors *.

ASFV Strain	Route	Dose	Infection	Reference
Georgia 2007	Drinking in media †	10^0^	37.5%	[51]
Georgia 2007	Drinking in media †	10^1^	44.4%	[51]
Armenia 2008	Oronasal	10^0.5^–10^1.4^ ‡	12.5%	[62]
Malawi 1983	Intraoropharyngeal	10^2^	0%	[63]
Malawi 1983	Intranasopharyngeal	10^2^	50%	[63]
Georgia 2007	Drinking in media †	10^2^	75%	[51]
Dominican Republic 1979	Intranasal/oral	10^2.7^	0%	[64]
Georgia 2007	Eating in complete feed †	10^3^	0%	[51]
Malta 1978	Intranasal	10^3^	60%	[65]
Georgia 2007	Drinking in media †	10^3^	83.3%	[51]
Netherlands 1986	Intranasal	10^3.5^	60%	[65]
Dominican Republic 1979	Intranasal/oral	10^3.7^	12.5%	[64]
East African	Eating in liquid or moistened solid feed †	10^3.7^–10^6.1^	0%	[66]
East African	Intranasal	10^3.7^–10^3.9^	100%	[66]
Tanzania KWH/12	Drinking in cow milk †	10^4^	0%	[67]
Georgia 2007	Eating in complete feed †	10^4^	40%	[51]
Malta 1978	Intranasal	10^4^	100%	[65]
Georgia 2007	Drinking in media †	10^4^	100%	[51]
Malawi 1983	Intranasopharyngeal	10^4^	100%	[63]
Malawi 1983	Intraoropharyngeal	10^4^	100%	[63]
Georgia 2007	Eating porcine plasma in complete feed †	10^4.3^	0%	[68]
Brazil 1978	Intranasal	10^4.5^	100%	[65]
Dominican Republic 1979	Intranasal/oral	10^4.7^	87.5%	[64]
Georgia 2007	Eating soft ticks in brioche †	10^4^–10^5^	33%	[69]
Georgia 2007	Eating porcine plasma in complete feed †	10^5^	0%	[68]
Unknown	Eating infectious material †	10^5^ ¶	MID	[70]
Tanzania KWH/12	Drinking in cow milk †	10^5^	37.5%	[67]
Georgia 2007	Eating in complete feed †	10^5^	44.4%	[51]
Tengani	Intranasal	10^5^	100%	[70]
Georgia 2007	Eating in brioche †	10^5.5^	0%	[69]
Dominican Republic 1979	Intranasal/oral	10^5.7^	90%	[64]
Georgia 2007	Eating in complete feed †	10^6^	25%	[51]
Tanzania KWH/12	Drinking in cow milk †	10^6^	75%	[67]
Malawi 1983	Intranasopharyngeal	10^6^	100%	[63]
Malawi 1983	Intraoropharyngeal	10^6^	100%	[63]
Georgia 2007	Eating soft ticks in brioche †	10^6^–10^7^	100%	[69]
Georgia 2007	Eating in complete feed †	10^7^	40%	[51]
Tanzania KWH/12	Drinking in cow milk †	10^7^	100%	[67]
Hinde WH II	Oral	10^7^–10^7.5^	100%	[71]
Hinde WH II	Eating in dry feed †	10^7^–10^7.5^	100%	[71]
Tengani	Intranasal	10^7.2^	100%	[72]
Georgia 2007	Eating in complete feed †	10^8^	50%	[51]
Tanzania KWH/12	Drinking in cow milk †	10^8^	100%	[67]
Kenya	Eating feces or urine contaminated feed †	ND	100%	[14]
Kenya	Eating in sweet potatoes or bananas †	ND	0%	[14]

* Studies are in ascending order as per the dose administered, with unknown doses at the end of the table. Within infection studies evaluating the same dose, references are in ascending order based on percentage infection. Dose shown as either 50% tissue culture infectious dose (TCID_50_) or 50% hemadsorbing dose (HAD_50_), except where indicated. † Indicates consumption through natural drinking or eating behaviors; ‡ dose shown in hemadsorbing units (HAU); ¶ unknown units used for quantification of dose. Key: MID, minimum infectious dose; ND, not determined.

**Table 2:** Studies reporting the efficacy of various feed additives in mitigating the risk of porcine viruses in feed.

Pathogen	Mitigant Composition	Inclusion Rate	Testing Method	Outcome or Effects	Reference
ASFV	Aqueous formaldehyde and propionic acid, MCFA (C6:C8:C10)	0.03–1.0%	Cell culture; tested in complete feed and ingredients at mean 12.3 °C temperature in a 30-day transoceanic shipment model	Dose-dependent inactivation; reduced infectivity in feed ingredients; decreased viral DNA quantity in feed ^a^	[94]
ASFV	MCFA (C8:C10:C12); GML	0.25–2.0%	Cell culture; complete feed stored for 30 min or 1 day at room temperature	Decreased virus titers in cell culture by MCFA and GML; dose-dependent antiviral activity by GML; reduced infectivity in complete feed by GML at ≥1.0%; no effect on viral DNA	[95]
PRRSV, SVA, PEDV	Aqueous formaldehyde, organic acids, acidifiers, HMTBa, SCFA, MCFA, LCFA, GML, and essential oils	0.1–3.0%	Ingestion of complete feed via natural consumption for 15 days	Decreased clinical signs and virus detection in biological samples; increased weight gain ^b^	[96]
PEDV	Lactic acid	0.75–1.5%	Stored in complete feed at 20 °C for 1 day	Reduced infectivity in feed	[97]
PEDV	Aqueous formaldehyde and propionic acid	0.33%	Stored in complete feed and ingredients under winter conditions (mean temperature between −9 °C and −18 °C) for 30 days	Reduced infectivity in feed and ingredients; decreased viral RNA quantity in feed	[98]
PEDV	Aqueous formaldehyde and propionic acid; MCFA (C6:C8:C10)	0.36–11.1%	Tested in rice hulls flushed through feed manufacturing equipment after complete feed	Decreased viral RNA quantity in rice hull flush ^a^	[99]
PEDV	Benzoic acid and essential oils	0.02–0.5%	Stored in complete feed for 42 days	Decreased viral RNA quantity in feed on combination treatment with both additives; no effect on virus infectivity in feed	[100]
PEDV	Aqueous formaldehyde and propionic acid; MCFA (C6:C8:C10)	0.33–2.0%	Tested in complete feed and ingredients at a mean 6.1 °C temperature in a 37-day transoceanic shipment model	Reduced infectivity in feed ingredients; decreased viral RNA quantity in feed ^a^	[41]
PEDV	Aqueous formaldehyde and propionic acid	0.32%	Ingestion of complete feed via natural consumption for 14 days	Prevented transmission to pigs through contaminated feed; decreased viral RNA quantity in feed	[101]
PEDV	Organic acids, acidifiers, sucrose, and sodium chloride	0.2–0.4%	Stored in complete feed at 25 °C for 21 days	Increased rate of virus decay in complete feed ^c^	[89]
PEDV	Aqueous formaldehyde and propionic acid; MCFA (C6:C8:C10)	0.125–1.0%	Stored in complete feed at room temperature for 1 day	Reduced infectivity in feed; dose-dependent decreased viral RNA quantity in feed	[102]
PEDV	MCFA (C6:C8:C10)	0.25–1.5%	Stored in complete feed at a mean 25.8 °C temperature for 40 days pre-inoculation; stored at room temperature for 3 days post-inoculation	Dose-dependent decreased viral RNA quantity in feed	[103]
PDCoV	Organic acids, acidifiers, sucrose, and sodium chloride	Low: 0.2–3.0% High: 0.4–6.0%	Stored in complete feed at 25 °C for 35 days	No effect at lower concentrations; increased rate of virus decay in complete feed at higher concentrations ^d^	[104]

Key: ASFV, African swine fever virus; PEDV, porcine epidemic diarrhea virus; PRRSV, porcine reproductive and respiratory syndrome virus; SVA, Seneca virus A; SCFA, short-chain fatty acids; MCFA, medium-chain fatty acids; LCFA, long-chain fatty acids; GML, glycerol monolaurate; HMTBa, methionine hydroxyl analogue. ^a^ Decreased nucleic acid quantity only associated with aqueous formaldehyde or high MCFA (11.1%) treatment; ^b^ improved outcome not seen with Vigilex (oils, fermentation products, whey products, plant protein) treatment; ^c^ increased rate of PEDV decay not seen after sodium chloride or Ultracid P (orthophosphoric, citric, fumaric, malic acids) treatment; ^d^ increased rate of PDCoV decay not seen after treatment with high concentrations of sucrose or formic acid.
